# The impact of psychotic experiences in the early stages of mental health problems in young people

**DOI:** 10.1186/s12888-018-1767-y

**Published:** 2018-06-28

**Authors:** Kareen Heinze, Ashleigh Lin, Barnaby Nelson, Renate L. E. P. Reniers, Rachel Upthegrove, Latoya Clarke, Ayesha Roche, Angelique Lowrie, Stephen J. Wood

**Affiliations:** 1 School of Psychology, University of Birmingham, Edgbaston, Birmingham, UK; 2 Institute for Mental Health, University of Birmingham, Edgbaston, Birmingham, UK; 3 Telethon Kids Institute, Perth, Australia; 4 Orygen, the National Centre of Excellence in Youth Mental Health, Melbourne, Australia; 5 Centre for Youth Mental Health, University of Melbourne, Melbourne, Australia; 6 Institute of Clinical Sciences, University of Birmingham, Edgbaston, Birmingham, UK; 7 Warwick Medical School, University of Warwick, Coventry, UK; 8 Department of Psychology, University of Sheffield, Sheffield, UK

**Keywords:** Clinical staging, Youth mental health, Psychotic experiences, Ultra-high risk

## Abstract

**Background:**

Anxiety and depressive symptoms and psychotic experiences constitute common features of emerging mental disorders in young people. Psychotic experiences and the ultra-high risk (UHR) state for psychosis appear to have a particular importance for clinical presentation, progression of symptomatology, quality of life and functioning, but the impact of psychotic experiences in individuals seeking help at non-UHR services, compared to UHR services, is under-researched.

**Methods:**

Sixty-nine young people (M_age_ ± SD at baseline = 20.8 ± 2.6, range 16–26 years, 48 females) presenting to mental health services were grouped according to UHR and non-UHR status. They were assessed at baseline for anxiety and depressive symptoms, psychological distress, psychosocial functioning and quality of life. They were followed up at three, six, and 12 months. Data were analysed using mixed linear modelling.

**Results:**

UHR individuals reported higher levels of depressive symptoms and psychological distress, and lower levels of role functioning and quality of life compared to non-UHR individuals. No differences were reported for anxiety symptoms or social functioning. Decline in psychosocial functioning was not associated with clinical deterioration or reduction of quality of life.

**Conclusions:**

Psychotic experiences appear to be particularly associated with depressive symptoms and psychological distress, impaired role functioning and quality of life in help-seeking young people in the medium-term. It is therefore important to pay special attention to psychotic experiences in the early stages of mental health problems even if psychotic symptoms are not the main motivation for help-seeking.

## Background

Mental illness is the major health problem experienced by young people [1, 2]. While some individuals in this age group have transient problems, many cases persist into middle and older adulthood and can severely impair psychosocial functioning. Adolescents and young adults often present in a non-specific way, with symptoms of depression and anxiety and psychotic experiences in the early stage of many longer term mental disorders [3]. Predicting who will have recurrent or chronic problems can be difficult, especially in the early stages of disorder.

One well researched example of early stage mental health problems is the ultra-high risk (UHR) state for psychosis. This refers to a presentation in which a young person experiences positive psychotic symptoms in a sub-threshold or transient form [4]. UHR individuals experience low psychosocial functioning and may have comorbid psychiatric disorders such as major depression [5, 6]. They frequently have poor functional outcomes [7], and are at increased risk of the onset of psychotic disorders [8] as well as non-psychotic disorders in the long-term [9, 10].

Psychotic experiences are not only associated with psychotic disorders, but are also prevalent in non-psychotic psychiatric illnesses such as depressive and anxiety disorders [11–13]. It has been hypothesised that psychotic experiences are not only a specific risk factor for developing psychosis, but can be a general marker of risk for a range of mental disorders [10, 14]. Indeed, in two population studies they have been found to be a risk factor for severe psychopathology more generally, not just psychotic disorder, characterised by high comorbidity and suicidal behaviour [15, 16].

This idea of an overlap between psychotic experiences and depressive and anxiety symptoms suggests the utility of a dimensional approach such as clinical staging. Using a clinical staging model, mental disorders are considered as dynamic syndromes that overlap and share aetiologies and courses [17], developing from a state of undifferentiated general symptoms and syndromes into more defined clinical conditions [3]. There is also evidence for bifactor [18] and unitary models including the “p-factor” model [19] which propose a general distress or psychopathology factor that underlies most common psychiatric disorders. Indeed, many risk factors, such as genetic disposition, trauma, and stressful life events, are shared between psychiatric illnesses [20].

The criterion of 30% decline in functioning or chronic low functioning has been added to UHR criteria in an attempt to increase the specificity of prediction of psychosis [21]. However, the European Prediction of Psychosis study (EPOS) identified a substantial loss of sensitivity in transitions when this drop in functioning was incorporated [22]. Except for the purpose of an enrichment strategy that theoretically predicts transition, the inclusion of this drop in functioning may be too restrictive when considering individuals’ overall psychopathology and too much focus on this functioning criterion risks missing cases who will also transition to psychosis [23].

The aims of the current study were two-fold. Firstly, we aimed to ascertain the role of positive psychotic experiences in individuals who were seeking help for common, non-psychotic mental health problems. It was hypothesised that those at UHR would cross-sectionally and longitudinally experience greater impairment in terms of higher psychopathology, poorer functioning and lower levels of self-reported quality of life compared to those identified as non-UHR.

Secondly, we investigated the role of functioning [21] added to the UHR criteria with regards to its utility in predicting clinical outcome and quality of life. We hypothesised that those with psychotic experiences but no functional decline and/or chronic low functioning would not significantly differ from those at UHR for psychosis (i.e. those with psychotic experiences and functional decline and/or chronic low functioning) on measures of psychotic and non-psychotic outcomes and quality of life in the medium term.

## Methods

### Participants & procedure

Seventy-three participants who were experiencing mental health problems took part in the baseline assessment of this study. They were recruited from the South Birmingham area of the United Kingdom via two clinical services (Youthspace & Birmingham Healthy Minds). Study inclusion criteria were being aged between 16 and 26 years and recent help-seeking (within 6 months of clinical contact) for mental health problems. Youthspace was a youth-focused secondary mental health service that provided support for young people (aged 16–25 years) with a variety of diagnoses and had no specific exclusion criteria, except for diverting individuals at UHR or with a first episode of psychosis to Early Intervention services in the South Birmingham area. Youthspace offered a variety of treatments and case management provided by a multi-disciplinary team. Birmingham Healthy Minds is a primary care psychological therapies service offering brief psychological therapy for individuals aged 16 and above who present with depressive and anxiety symptoms. Their exclusion criteria are bipolar disorder, psychosis, suicidality or need of long-term care. Both services operated primarily though general practitioner referral at the time of study recruitment. Exclusion criteria for the study were a lack of sufficient English and cognitive ability to provide informed consent and adequately complete assessments. The study was approved by the West Midlands - Edgbaston Research Ethics Committee (reference number: 12/WM/0135) and participants provided written informed consent.

Participants were either approached by their clinician during a clinical session or after a session by a member of the research team. Participants received some brief verbal information about the study as well as an information sheet and could either immediately arrange an appointment for the baseline assessment if approached by a member of the research team or sign a consent to be contacted form if approached by a service staff member. If the latter was the case, a member of the research team would contact the participant by telephone or via letter. A small number of participants were also recruited via advertisements at Youthspace and Birmingham Healthy Minds. Those participants were screened for meeting the inclusion criteria when getting in touch with the research team. Individuals who were scheduled for a baseline assessment, received a detailed participant information sheet before they gave informed written consent. Interview and questionnaire assessments were administered by trained members of the research team (KH, AL, LC, AR, AL, KC, HB, CS, EW, CB). Most assessments were conducted in facilities of the University of Birmingham. In rare cases home visits were conducted or follow-up interviews were administered via telephone, if it was not possible for the participant to come in.

80.8% (*n* = 59) of these individuals provided data for the 3 month follow-up assessment, 75.3% (*n* = 55) for the 6 month and 53.4% (*n* = 39) for the 12 month follow-up assessment. 46.6% (*n* = 34) of individuals provided data for all three follow-up assessments, 26% (*n* = 19) for two follow-up assessments, 16.4% (*n* = 12) for one follow-up assessment and 11% (*n* = 8) for none of the follow-up assessments.

### Interventions

Clinical care for participants included psychotropic medication and/or psychological therapies/counselling: of those participants who were assessed at baseline, 50.7% (*n* = 37) were taking antidepressant medication and an additional 5.5% (*n* = 4) were prescribed antipsychotic and/or mood stabilising medication. Additionally, 52.1% (*n* = 38) of participants were receiving counselling or some sort of therapy (e.g. cognitive-behavioural or -analytic therapy) and 32.9% (*n* = 24) had been assessed or referred for therapy.

### Measures

Participants completed an interview and self-report assessment on the following measures at all four time points:

#### Comprehensive Assessment of At-Risk Mental States (CAARMS)

The CAARMS [4] is a semi-structured interview designed to determine the at-risk mental state for psychosis. The four subscales unusual thought content (e.g. delusional mood, overvalued ideas), non-bizarre ideas (e.g. suspiciousness, grandiosity), perceptual abnormalities (e.g. distortions, illusions, hallucinations), and disorganised speech (e.g. difficulties with speech and communication) quantify severity (0 = absent/never-6 = psychotic and severe) and frequency (0 = absent/never-6 = continuous) of psychotic experiences. A combination of intensity and frequency ratings allows for the determination of whether individuals meet criteria for being at UHR for psychosis and for determining onset of first episode psychosis (FEP). A score of at least three for both intensity and frequency on at least one subscale (with exception of at least four for intensity for disorganised speech) indicates UHR status, if coupled with a decline in functioning or chronic low functioning. The CAARMS indicates UHR status if symptoms were present over the last 12 months. An overall inter-rater reliability of 0.85 has been reported and CAARMS criteria displayed good concurrent (e.g. with the Brief Psychotic Rating Scale) and predictive validity (e.g. higher risk of transition to psychosis in individuals with an at-risk mental state) [4].

Criteria for FEP are met if participants score a 6 on intensity and at least a 4 for frequency on non-bizarre ideas, unusual thought content or disorganised speech or a 5–6 on intensity and a 4–6 on frequency for perceptual abnormalities.

#### Social and Occupational Functioning Assessment Scale (SOFAS)

The SOFAS has been derived from the Global Assessment Scale [24] which is a modestly reliable and valid measure of psychiatric disturbance [25], and provides a rating of overall psychological functioning on a scale from 0 to 100 [26]. The SOFAS is usually used to rate an individual’s current functioning, however highest and lowest functioning ratings for the past 12 months were employed to determine a drop in functioning. The researcher rated the score on the SOFAS based on information provided in the interview for the Global Functioning: Social and Role Scales. The SOFAS has been included in the Diagnostic and Statistical Manual for Psychiatric Disorders IV-TR [27] to overcome short-comings of existing measures of individuals’ functioning [28].

#### Classification based on psychotic experiences

At each time point participants were classified into individuals at UHR for psychosis as opposed to those who did not fulfil UHR criteria (“non-UHR”). A further distinction was made concerning the relevance of the functioning criterion for the definition of UHR status: individuals with psychotic experiences with both an intensity and frequency of at least three on the CAARMS, but without a 30% drop in functioning or chronic low functioning (SOFAS score ~ 50 during the past 12 months) were referred to as “psychotic experiences without functional decline” (as opposed to UHR who experienced either this described drop in functioning or chronic low functioning). All combinations of an intensity and frequency of less than three on all sub-scales were considered as “no significant psychotic experiences”, regardless of functioning. Hence, an additional three-group comparison was conducted with individuals at UHR, individuals with psychotic experiences without functional decline and individuals with no significant psychotic experiences.

#### Quick Inventory of Depressive Symptoms (QIDS)

The QIDS [29] is a 16-item, semi-structured interview to gauge severity of depressive symptoms over the past 7 days. Items are scored 0–3 and total scores range from 0 to 27. A meta-analysis reported concurrent validity, e.g. with the Hamilton Rating Scale for Depression ranging from 0.72 to 0.79 and a Cronbach’s α ranging from 0.65 to 0.87 [30]. Cronbach’s α in this sample ranging from 0.61 to 0.78 across time points.

#### Overall Anxiety Severity and Impairment Scale (OASIS)

The OASIS [31] is a brief five-item self-report questionnaire of severity and impairment across multiple anxiety disorders and sub-threshold anxiety. It captures frequency and intensity of anxiety, avoidance behaviour and interference of anxiety with everyday life and relationships. Total scores range from 0 to 20. The OASIS showed convergence with major anxiety measures (e.g. for social, posttraumatic stress, and generalised anxiety), a Cronbach’s α of 0.84 for the five items [31], and one-month re-test reliability of 0.82 [32].

#### Kessler psychological distress scale (K-10)

The K-10 [33] is a 10-item self-report questionnaire assessing psychological distress via questions about depressive and anxiety symptoms in the past 30 days. Items are rated on a five-point scale with total scores ranging from 10 to 50. The K-10 is a moderately reliable instrument (kappa ranging from 0.42 to 0.74) [34] and demonstrates good concurrent validity with other instruments such as the General Health Questionnaire and current diagnosis of anxiety and affective disorders [35]. Cronbach’s α in this sample ranged from 0.88 to 0.89 across time points.

#### Psychosocial functioning

The Global Functioning: Social [36] and Role [37] Scales are semi-structured interviews and were used to index current social and role functioning, providing overall scores from 1 to 10, with 10 indicating superior functioning and 1 extreme dysfunction. Inter-rater reliability ranged from 0.85 to 0.95, and the social functioning scale was significantly correlated with social contacts (*r* = 0.70) and role functioning with work and school functioning (*r* = 0.57) [38].

#### Quality of life

Perceived quality of life was assessed using the World Health Organisation Quality of Life (WHOQoL) [39] self-reported item “Thinking about your life in the last four weeks, how would you rate your quality of life”, on a five-point scale from 1 (“very poor”) to 5 (“very good”) retrieved from the 26-item measure WHO-BREF. All four domains of quality of life of the WHO-BREF correlated significantly with this item, whereby psychological and environmental domains were more strongly associated as compared to social and physical domains. This indicates that the item that we used not only demonstrated face validity but also represents the WHO-BREF, which is characterised by good to excellent psychometric properties, well [40].

### Statistical analysis

An independent samples t-test was used to compare UHR and non-UHR individuals for age. For analysis of categorical data, such as gender, ethnicity, occupation and highest education, χ^2^ tests were used to evaluate group differences. To increase robustness of findings, binary linear regression analyses were conducted to identify whether or not attending any of the three follow-up assessments was related to high or low scores in any of the clinical or functioning measures or quality of life at the respective prior assessment.

As there was incomplete data at all four time points (34 individuals who completed all assessments), mixed linear modelling was implemented using four time points (baseline, three, six and 12 months) for both the UHR vs non-UHR comparison and classification of no significant psychotic experiences, UHR, and psychotic experiences without functional decline for the dependent variables QIDS, OASIS, K-10, social and role functioning, and WHOQoL score. Group and time (and their interaction) were specified as fixed effects in the model as we assumed that individual-specific effects were correlated with both factors. Bonferroni post-hoc tests were used to compare QIDS, K-10, OASIS, and WHOQoL scores between UHR, individuals with psychotic experiences without functional decline and no significant psychotic experiences within the mixed linear modelling analyses.

## Results

### Demographics and UHR vs non-UHR group comparisons at baseline

At baseline, 56.2% (*n* = 41) of participants presented with no significant psychotic experiences, 19.2% (*n* = 14) were experiencing psychotic experiences without functional decline, whereas another 19.2% (*n* = 14) of individuals were at UHR. Four participants fulfilled CAARMS criteria for a FEP (5.5%) and were therefore excluded from the remainder of the study, leaving a final sample of 69 participants. Out of the final sample of 69 individuals, the 14 individuals with psychotic experiences without functional decline (20.3%) and 41 individuals with no significant psychotic experiences (59.4%) formed the non-UHR group, as opposed to the UHR group (*n* = 14; 20.3%).

Transition to FEP according to CAARMS rating was monitored and recorded for two participants at the three-month follow-up assessment and an additional participant at the six-month follow-up assessment for those individuals who took part in the respective assessments. No further transitions were recorded for the 12-month follow-up assessment (overall transition rate of 3/69 of whole sample: 4%; overall transition rate of 3/15 individuals identified as UHR at baseline: 27%).

The total sample (*n* = 69, 48 females, 69.6%) had a M_age_ ± SD of 20.8 ± 2.6 years and was predominantly White-British. At baseline, there were no significant group differences on demographic variables between UHR and non-UHR individuals (see Table 1).

**Table 1:** Baseline demographic information for the total sample, and comparing UHR and non-UHR at baseline

	Total sample (*n* = 69)	UHR (*n* = 14)	Non-UHR (*n* = 55)	Test statistics
*Age* (M ± SD) in years	20.8 ± 2.6	20.8 ± 3.1	20.7 ± 2.5	t (67) = 0.12, *p* = 0.91
Gender (m/f)	21/48	5/9	16/39	X ^2^ (1) = 0.23, *p* = 0.63
Ethnicity				
White^a^	58	12	46	
Asian^b^	3	1	2	
Black^c^	2	0	2	X ^2^ (3) = 0.88, *p* = 0.83
Mixed-race^d^	6	1	5	
Occupation				
University student^e^	18	1	17	
College/A-Levels	20	3	17	
Unemployed	11	2	9	X ^2^ (4) = 7.70, *p* = 0.10
Employed^f^	17	7	10	
Homemaker	3	1	2	
Highest qualification				
University^g^	6	1	5	
A-Levels^h^	31	4	27	X ^2^ (3) = 2.91, *p* = 0.41
GSCE^i^	26	8	18	
No qualification	6	1	5	

*UHR* ultra-high risk*, M* mean, *SD* standard deviation, *m* male, *f* female

^a^White-British & White-Other

^b^Asian-Pakistani, Asian-Bangladeshi & Other Asian

^c^Black-African

^d^Mixed-Race White-Black-Caribbean

^e^Undergraduate and postgraduate university students

^f^Working full or part-time

^g^Bachelor or Master degree

^h^A-Levels, National Vocational Qualification (NVQ) Level 4, or equivalent

^i^General Certificate of Secondary Education (GCSE, year-10 equivalent) or NVQ level 1 or 2

None of the clinical or functioning measures or quality of life were associated with non-attendance at three, six and 12 months follow, except for lower OASIS scores at 3 month follow-up that predicted non-attendance at 6 month follow-up (6 month follow-up attendance, *n* = 45, OASIS M ± SD: 7.9 ± 3.9; 6-months follow-up non-attendance, *n* = 6, OASIS M ± SD: 3.3 ± 3.1; *p* = 0.036).

### Longitudinal UHR vs non-UHR group comparisons

Statistical comparisons revealed that there were no differences between those individuals that were included in the baseline analyses and those who did not take part at three, six or 12 month follow-up concerning any clinical baseline measures or classification as UHR and non-UHR (all *p* > 0.05). UHR vs non-UHR classification at three, six, and 12-month follow-up was conducted in accordance with procedure of baseline classification, yet with ratings from each respective assessment. Due to the small number of transitions (*n* = 3) and the fact that transition to FEP was determined on the basis of the CAARMS as a classification tool and not on clinical criteria or eligibility for treatment with early intervention services, data was handled as “non-UHR” in all three follow-up cases due to no drop in or chronic low functioning (and as “psychotic experiences without functional decline” for the three-group comparison in 3.3). However, additional mixed linear modelling was conducted that excluded the three transitioned cases, to investigate robustness of findings.

**Fig. 1:**
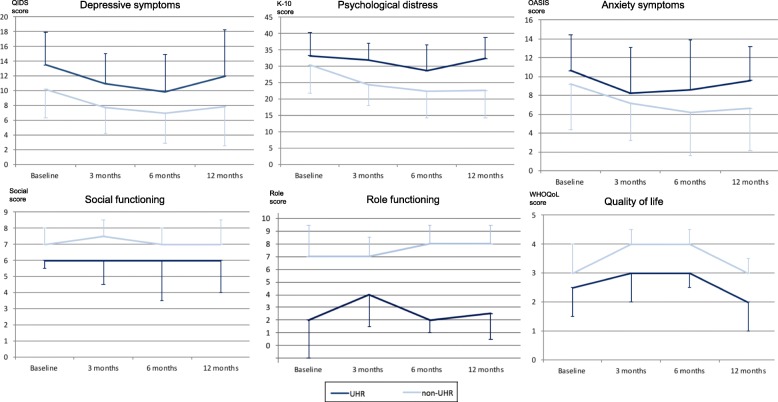
Clinical measures, functioning and quality of life for UHR and non-UHR over time. QIDS = Quick Inventory of Depressive Symptoms, K-10 = Kessler Psychological Distress Scale, OASIS=Overall Anxiety Severity and Impairment Scale, Social = social functioning, Role = role functioning, WHOQoL = Quality of life, UHR = ultra-high risk; central tendency displayed as mean +/− standard deviation for depressive symptoms, psychological distress and anxiety symptoms and as median +/− interquartile range for social and role functioning and quality of life scores

Ten individuals were classified as UHR at 3 month (16.9%), nine at 6 month (16.3%) and ten at 12-month follow-up (25.6%). Three individuals (4.3%) were classified as UHR at all their assessments, 44 (63.8%) were classified as non-UHR at all their assessments, and 22 (31.9%) changed UHR status at least once during the follow-up period. Linear mixed effects modelling was conducted with time (baseline, three, six and 12 months) and group (UHR vs non-UHR) factors, where group affiliation allowed for change across the four time points (e.g. from non-UHR at baseline to UHR at 3 months or vice versa). Analyses revealed significant group differences between UHR and non-UHR across the four time points for the following: QIDS (F (1, 132.26) = 15.27, *p* < 0.001), K-10 (F (1, 114.91) = 12.64, *p* = 0.001), role functioning (F (1, 159.87) = 21.52, *p* < 0.001) and WHOQoL (F (1, 160.54) = 7.68, *p* = 0.006). UHR individuals demonstrated overall higher QIDS scores, and K-10 scores and lower role functioning and WHOQoL scores. Significant time effects were found for the QIDS (F (3, 65.30) = 6.43, *p* = 0.001), OASIS (F (3, 59.54) = 5.58, *p* = 0.002), K-10 (F (3, 54.44) = 5.48, *p* = 0.002) and WHOQoL (F (3, 79.72) = 2.87, *p* = 0.042) indicating a tendency for improvement in symptomatology and quality of life over time in the group as a whole. No interaction effects were found. When analyses were repeated without the three cases who transitioned to psychosis over the follow-up period, results from linear mixed effects modelling remained qualitatively very similar, except that all significant time effects disappeared. Measures of central tendency for QIDS, OASIS, K-10, social and role functioning and WHOQoL scores across the four time points between UHR and non-UHR individuals are illustrated in Fig. 1.

### Classification into UHR, psychotic experiences without functional decline and no significant psychotic experiences from baseline to follow-up

Statistical comparisons revealed that there were no differences between those individuals that were included in the baseline analyses and those who did not take part at three, six or 12 month follow-up concerning any clinical baseline measures or classification as UHR, psychotic experiences without functional decline and no significant psychotic symptoms (all *p* > 0.05).

**Fig. 2:**
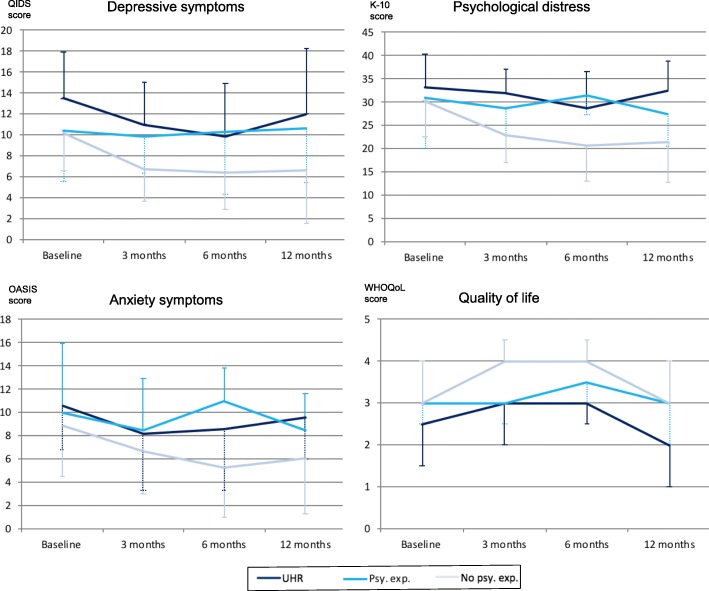
Three-group comparison of clinical measures and quality of life over time. QIDS = Quick Inventory of Depressive Symptoms, K-10 = Kessler Psychological Distress Scale, OASIS=Overall Anxiety Severity and Impairment Scale, UHR = ultra-high risk for psychosis, psy. exp. = psychotic experiences without functional decline, no psy. exp. = no significant psychotic experiences; central tendency displayed as mean +/− standard deviation for depressive symptoms, psychological distress and anxiety symptoms and as median +/− interquartile range for quality of life scores

Fourteen (23.7%) individuals were classified as psychotic experiences without functional decline at baseline, 13 (22.0%) at 3 month, 6 (10.9%) at 6 month and nine (23.1%) at 12-month follow-up. Overall, there was a variety of trajectories concerning the three-group classification from baseline and across the three follow-up time points, with the majority of individuals presenting as UHR or psychotic experiences without functional decline for at least one time point (*n* = 42, 60.9%). Linear mixed effects modelling revealed significant group differences between individuals with no significant psychotic experiences, UHR and psychotic experiences without functional decline across the four time points for the following: QIDS (F (2, 125.48) = 11.90, *p* < 0.001), OASIS (F (2, 123.99) = 4.69, *p* = 0.011), K-10 (F (2, 97.22) = 11.14, *p* < 0.001), and WHOQoL (F (2, 157.77) = 6.51, *p* = 0.002). Time effects were found for QIDS (F (3, 63.63) = 5.82, *p* = 0.001), OASIS (F (3, 58.38) = 3.73, *p* = 0.016), K-10 (F (3, 53.15) = 3.99, *p* = 0.012) and WHOQoL (F (3, 79.50) = 3.22, *p* = 0.027). No interaction effects were found. When analyses were repeated without the three cases that transitioned over the follow-up period, results from linear mixed effects modelling remained similar. Figure 2 illustrates this three-group comparison for QIDS and OASIS, K-10, and WHOQoL at all four time points.

Bonferroni post hoc tests indicated a difference between those with no psychotic experiences as compared to those with psychotic experiences without functional decline and as compared to UHR individuals (except for OASIS score between those with no psychotic experiences and UHR individuals and for WHOQoL score between those with and without psychotic experiences), but no differences between individuals at UHR as compared to those with psychotic experiences without functional decline (see Table 2).

**Table 2:** Bonferroni post-hoc tests for three-group comparison

Measure Contrast	Bonferroni test *p*-value
QIDS	
No psy. exp. vs psy. exp.	0.011^*^
No psy. exp. vs UHR	< 0.001^***^
Psy. exp. vs UHR	0.639
K-10	
No psy. exp. vs psy. exp.	0.029^*^
No psy. exp. vs UHR	< 0.001^***^
Psy. exp. vs UHR	0.664
OASIS	
No psy. exp. vs psy. exp.	0.015^*^
No psy. exp. vs UHR	0.153
Psy. exp. vs UHR	1.00
WHOQoL	
No psy. exp. vs psy. Exp.	0.251
No psy. exp. vs UHR	0.002^**^
Psy. exp. vs UHR	0.427

*QIDS* Quick Inventory of Depressive Symptoms, *OASIS* Overall Anxiety Severity and Impairment Scale, *K-10* Kessler Psychological Distress Scale, *no psy. exp* no significant psychotic experiences, *psy. exp* psychotic experiences without functional decline, *UHR* ultra-high risk for psychosis

^*^p < 0.05, ^**^p < 0.01, ^***^p < 0.001

## Discussion

In the present study we examined the role of psychotic experiences in predicting current and future psychopathology, psychosocial functioning and quality of life in a sample of young, help-seeking individuals with mental health problems. Although participants were recruited from general (not UHR-specific) services, one fifth were classified as UHR for psychosis and an additional one fifth had significant psychotic experiences without functional decline at entry to the study, comparable to the rate in similar studies (e.g. [41]). Mostly consistent with our hypotheses, UHR individuals reported higher levels of depressive symptoms and psychological distress, and lower levels of role functioning and quality of life compared to non-UHR individuals. As opposed to our hypotheses, no differences were reported for anxiety symptoms or social functioning. When we explored the predictive value of the functioning criterion for definition of UHR status, as predicted, there were no significant differences in all measures between individuals at UHR and those with psychotic experiences without functional decline.

There were no group differences between UHR and non-UHR individuals on anxiety symptoms, and social functioning, although the UHR group showed significantly higher depressive symptoms and substantially lower levels of role functioning than non-UHR individuals. This may likely be explained by the nature of the UHR status and the drop in functioning or chronic low functioning being driven by low levels of role functioning. It is possible that low role functioning is particularly characteristic for this sample comprising of UHR individuals not specifically help-seeking for psychotic experiences but for general mental health problems. Most UHR studies report psychosocial functioning combined (e.g. [10, 21, 22]), however, studies that examined social and role functioning separately using the same measure, found similar levels of social functioning and higher levels of role functioning as compared to the current study (e.g. [38]). Role functioning may therefore be a particularly important target for early intervention, for example, in terms of vocational and educational interventions in addition to symptom-oriented psychotherapy. Vocational rehabilitation was found to be effective in chronic schizophrenia [42], and has recently been introduced to individuals with a FEP in a randomised controlled trial [43], yet there may be a need for such interventions even earlier in the course of illness.

The finding of higher depressive symptoms and psychological distress, and lower role functioning and quality of life in UHR individuals as compared to non-UHR individuals is in accordance with Wigman et al. [44] who found that clients with non-psychotic psychiatric disorders but additional psychotic experiences showed lower global functioning than those without psychotic experiences. Psychotic experiences have also been shown to predict depressive symptoms in the future [45], yet it has to be acknowledged that there is also contrary evidence indicating a cross-sectional association only [13]. These findings are also consistent with the idea that psychotic experiences are associated with more severe mental health problems [12, 46].

UHR status was assessed at each follow up assessment and not only at baseline. This is especially important considering the dynamics of clinical presentation. Indeed, almost one third of individuals changed from UHR to non-UHR (or vice versa) at least once over the follow-up period of only 12 months. This indicates that UHR is not a static concept and clinicians should be aware of the potential for rapid changes in symptomatology.

A considerable amount of the UHR literature has focused on transition to psychosis as the primary outcome. However, it is not only the prediction of transition to psychosis that is key to ensuring young people with mental health problems receive the care they need – focusing on other psychopathology is equally important considering outcomes of those individuals who do not transition to psychosis. Lin et al. [10] followed non-transitioned UHR individuals and found that more than two thirds experienced non-psychotic disorders over the follow-up period of up to 14 years, with 90% presenting with non-psychotic disorders at baseline. This study supports a shift of emphasis from categorical outcomes (e.g. transition to psychosis or assignment of clinical diagnosis) to a more holistic approach of mental health outcomes. In the current sample only 4% transitioned to psychosis, but many experienced ongoing significant psychopathology.

This current study illustrated the distribution of clinical symptoms and quality of life of individuals at UHR and those with psychotic experiences without functional decline and participants with no significant psychotic experiences. Post-hoc tests revealed no significant differences between individuals at UHR and those with psychotic experiences without functional decline. Thus we found no evidence for an exacerbation in clinical symptomatology if functional decline or chronic low functioning was present. Whereas the inclusion of functional decline for UHR status definition serves its purpose to increase specificity of prediction of psychosis [21], it may not be a robust marker for prediction of clinical deterioration or a decrease in quality of life. However, the current results should be interpreted with caution considering the small group sizes.

Lastly, individuals who took part in this study were recruited from both a primary and secondary mental health service in the UK. It is plausible that individuals presenting to secondary mental health services are more impaired concerning their mental health and psychosocial functioning as those presenting to primary care services, considering that the UK operates on general practioner referral who are likely to have more conservative thresholds of what constitutes a psychiatric case as opposed to countries that operate on self-referral for mental health issues [47]. However, actual access to secondary care may be more driven by cost of and capacity for service provision than need for clinical care or illness severity: examination of transition protocols from child to adolescent mental health services revealed that only one quarter of cases that were deemed suitable for transition, actually ‘graduated’ to an adult mental health service, leaving a service gap especially for 16 and 17 year olds where mental health services are disproportionately expensive [48].

There were several limitations to the current study. The study was characterised by reasonably high attrition rates and the cohort was followed-up over only 12 months, although these first 12 months appear to be the most relevant time period, including the highest number of actual transitions to a FEP [49]. Although the sample was quite small (in particular the numbers of individuals at UHR and those with psychotic experiences without functional decline), the participants provided detailed and comprehensive psychopathological information. The sample comprised a very heterogeneous clinical presentation (including heterogeneous treatment and care setting for help-seeking which we were not able to control for in our analyses) with participants differing widely from none to severe symptom presentation across diagnostic categories. However, the presented findings are not fixed to diagnostic categories during the early stages of mental health, constituting a different approach to mental health that aims to circumvent issues around comorbidity [50] and the question of the existence of natural boundaries between mental disorders [51].

## Conclusions

The current study explored the role of psychotic experiences and being at UHR for psychosis in youth who were seeking help for common, non-psychotic mental health problems. Individuals at UHR for psychosis demonstrated significantly higher levels of depressive symptoms and psychological distress, and lower role functioning and quality of life, as compared to non-UHR individuals. Therefore, in addition to symptom-orientated psychotherapy, it may be important to also focus on individuals’ compromised role functioning, and consider using vocational and educational rehabilitation in these early stages of mental health problems. Lastly, functional decline and chronic low functioning did not exacerbate clinical symptomatology and may therefore not be a robust marker for prediction of clinical deterioration or a decrease in quality of life.

## Abbreviations

ANOVA: Analysis of variance
CAARMS: Comprehensive assessment of at-risk mental states
FEP: First episode of psychosis
K-10: Kessler psychological distress scale
OASIS: Overall anxiety severity and impairment scale
QIDS: Quick inventory of depressive symptoms
SOFAS: Social and occupational functioning assessment scale
UHR: Ultra-high risk
WHOQoL: World Health Organisation Quality of Life
